# Exercise-Induced Plasticity in Signaling Pathways Involved in Motor Recovery after Spinal Cord Injury

**DOI:** 10.3390/ijms22094858

**Published:** 2021-05-04

**Authors:** Jadwiga N. Bilchak, Guillaume Caron, Marie-Pascale Côté

**Affiliations:** Marion Murray Spinal Cord Research Center, Department of Neurobiology and Anatomy, Drexel University, Philadelphia, PA 19129, USA; jnb94@drexel.edu (J.N.B.); guillaume.caron@u-paris.fr (G.C.)

**Keywords:** exercise, rehabilitation, spinal cord injury, sprouting, regeneration, BDNF, serotonin, chloride homeostasis, KCC2

## Abstract

Spinal cord injury (SCI) leads to numerous chronic and debilitating functional deficits that greatly affect quality of life. While many pharmacological interventions have been explored, the current unsurpassed therapy for most SCI sequalae is exercise. Exercise has an expansive influence on peripheral health and function, and by activating the relevant neural pathways, exercise also ameliorates numerous disorders of the central nervous system (CNS). While the exact mechanisms by which this occurs are still being delineated, major strides have been made in the past decade to understand the molecular underpinnings of this essential treatment. Exercise rapidly and prominently affects dendritic sprouting, synaptic connections, neurotransmitter production and regulation, and ionic homeostasis, with recent literature implicating an exercise-induced increase in neurotrophins as the cornerstone that binds many of these effects together. The field encompasses vast complexity, and as the data accumulate, disentangling these molecular pathways and how they interact will facilitate the optimization of intervention strategies and improve quality of life for individuals affected by SCI. This review describes the known molecular effects of exercise and how they alter the CNS to pacify the injury environment, increase neuronal survival and regeneration, restore normal neural excitability, create new functional circuits, and ultimately improve motor function following SCI.

## 1. Introduction

Spinal cord injury (SCI) is a devastating condition and a major cause of disability, with up to half a million cases reported every year worldwide [1]. In the United States alone, there are an estimated 300,000 people living with a spinal cord injury (SCI), with approximately 17,000 new cases arising each year [1]. SCI is associated with substantial personal and societal costs, as well as secondary complications that often exceed the original injury and lead to a reduced quality of life and increased rates of mortality. While there is yet no definitive cure for SCI, many of its sequalae can be alleviated by exercise-based rehabilitation programs and activity-based therapies. In the clinic, the planned, structured, and repetitive physical activity produced by spinal networks and skeletal muscle has noticeable beneficial effects on an array of functional systems, including respiratory [2,3,4], cardiovascular [5,6] bladder, bowel, and sexual function [7,8]. In addition, by providing repetitive and relevant sensory cues, effective rehabilitation strategies have the potential to retrain the nervous system to improve performance of motor tasks such as walking and standing [9,10,11,12,13].

Rehabilitation approaches that promote repetitive motor activity are considered the gold-standard therapy and are widely used in the clinic as a critical component of successful functional recovery in SCI individuals [9,14,15]. The beneficial effects of exercise after SCI suggest that it provides sufficient excitation to the nervous system to promote the activity both of uninjured spinal pathways and of residual supraspinal inputs. While clinical studies are necessary to test whether specific strategies provide relief for individuals with SCI, these rely on evidence-based therapies [6,16], which do little to elucidate the mechanisms contributing to recovery [15]. Therefore, investigating activity-dependent plasticity in animal models is critical, not only to optimize rehabilitation programs in the clinic, but also to identify potential pharmacological strategies. These may be used either in place of exercise when it is not possible due to comorbidities or lack of access, or in conjunction with exercise to enhance functional improvements. It is thus imperative that the molecular underpinnings of exercise are understood in full in order to maximize its potential in the clinic.

There is abundant evidence that exercise facilitates motor recovery after SCI, but how an increase in physical activity leads to improvements in neurological function, especially in terms of molecular mechanisms that promote plasticity of the injured spinal cord, remains elusive. In this review, we compile and summarize how exercise affects signaling pathways in the spinal cord to enhance functional motor recovery after SCI. Together, the current literature suggests that exercise can: (1) modify the injury environment; (2) promote axonal sprouting of local spinal networks and remaining descending axons; (3) promote synaptic and ionic plasticity; and, importantly, (4) improve motor functions in both the hindlimbs and forelimbs, validating the therapeutic potential of task-specific rehabilitation for functional recovery after chronic SCI.

## 2. Effect of Rehabilitation on Spinal Networks below the Injury

### 2.1. Synaptic Plasticity and Synapse Formation

**Figure 1:**
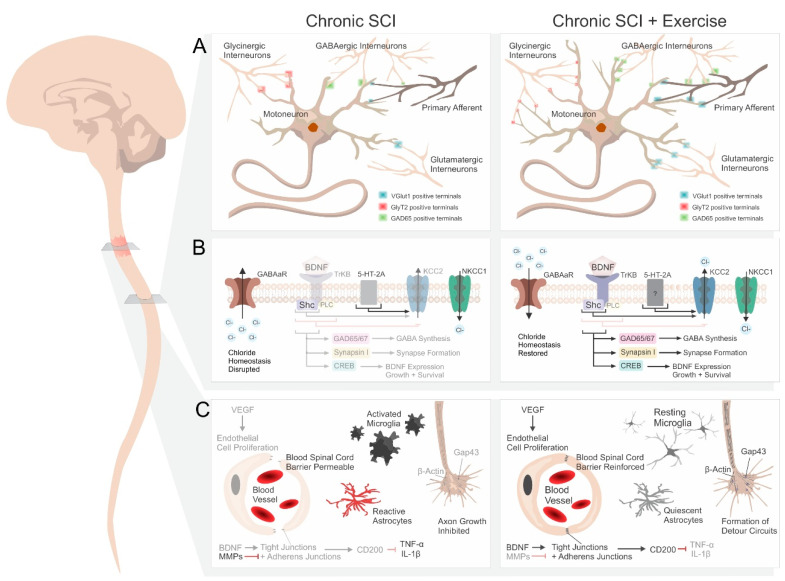
Schematic representation of various alterations induced by exercise following spinal cord injury (SCI). (**A**) Below the lesion, SCI causes atrophy of motoneuronal dendrites, reduced number of motoneuronal synapses, and less pre-synaptic inhibition of primary afferents. Exercise increases/maintains the number of synapses on motoneurons, as well as on primary afferents, with various changes in synaptic terminal size. (**B**) Many signaling pathways that ensue following SCI and exercise depend on BDNF signaling, including chloride homeostasis, neurotransmitter regulation, synapse formation, and neuronal growth and survival. The 5-HT2_A_R is believed to be upregulated after SCI, and further enhanced by exercise. (**C**) Within and around the lesion site, exercise increases angiogenesis, axon sprouting, and detour circuit formation, while reducing inflammation and glia reactivity.

Synaptic plasticity plays a pivotal role in functional recovery after SCI, and extensive molecular and morphological changes have been documented that implicate exercise as a reliable initiator of activity-dependent neuroplasticity. After a thoracic SCI, exercise causes lumbar spinal neurons below the injury to display an increase in: (1) the expression of synaptophysin [17,18] and Synapsin I [19], important presynaptic markers; (2) the expression of PSD-95 [17], a postsynaptic scaffolding protein; and (3) the number of VGlut1^+^ terminals, an indicator of glutamatergic synapses [20]. Together, these changes indicate an increased number of synaptic inputs below the lesion [21] (Figure 1A). While the relationship between the density of synaptic inputs and their efficiency cannot be determined based on anatomical data alone, these changes observed in the lumbar cord are associated with an improvement in stepping performance. Similarly, reaching and grasping training significantly increases Synapsin I around ventral horn motoneurons in the cervical spinal cord caudal to the lesion and is associated with improved reaching and grasping ability [22]. Overall, this suggests that by providing repeated activity within neural networks, exercise strengthens synaptic connections and either promotes synaptic formation and/or maintains synapses that would have otherwise degraded after injury. The neuronal populations from which these synaptic structures originate is uncertain, but their presence after a complete spinal cord transection suggests that this synaptic reorganization may originate from sensory afferents as well as a number of interneurons. Models of incomplete injuries also suggest a role for sprouting of spared supraspinal pathways (further discussed in Section 3).

There are several mechanisms by which exercise likely promotes synaptic plasticity. For example, exercise increases synthesis of the transcription factor cyclic AMP response element binding protein (CREB) and its phosphorylated form, pCREB, in the spinal cord caudal to the injury, in association with improved functional recovery [19,23] (Figure 1B). CREB is one of the best-characterized transcription factors and contributes to cell survival following insults to the CNS, as well as motor learning [24,25]. Another possible contributing factor is that exercise prevents the SCI-induced downregulation of perineuronal nets (PNNs) around lumbar motoneurons below the lesion site [20,26,27]. PNNs are formed of extracellular matrix molecules, i.e., chondroitin sulphate proteoglycans (CSPGs) that aggregate extracellularly on the surface of neuronal cell bodies and proximal dendrites [28]. The role of PNNs is complex and can be both protective and inhibitory; while they restrict axonal plasticity and limit the ability of the spinal cord to reorganize following injury, they are also involved in the fine-tuning of function by regulating ion buffering, neuroprotection, and synaptic stabilization, and by curtailing aberrant plasticity [29]. There is also strong evidence that PNNs are associated with activity-dependent synaptic plasticity during development [30] and after injury [31]. Although the effect of rehabilitation on PNNs remains to be fully described, recent studies have suggested that rehabilitation increases PNNs and the level of CSPGs in the lumbar spinal cord in intact animals [26] and in animals with SCI [27], in association with improved functional recovery. This suggests that the increased neuronal activity provided by exercise may prevent the loss of stabilizing structures and maintain existing synapses through an increase in expression of CSPGs and PNNs in the associated spinal circuits. There is also substantial evidence supporting an exercise-induced formation of new synapses. Exercise increases the growth-associated Protein 43 (GAP43) after SCI [19], which appears to act in concert with Synapsin I [32]. GAP43 is expressed at high levels in neuronal growth cones (Figure 1C), and plays a critical role in axon growth, regeneration, and the formation of new connections [33]. Additionally, large changes in morphology have been reported, implying that new circuits are formed.

SCI causes significant dendritic atrophy and synaptic stripping of motoneurons caudal to the lesion [18,34], and both treadmill training [18] and bike-training [35] were shown to increase dendritic density and total neurite length compared to sedentary controls. Upon closer examination, it was found that the size, density, and total number of various synapses on lumbar motoneurons of exercised animals with SCI differed substantially from those in intact animals [36]. This was accompanied by significant recovery of locomotor ability. This suggests, perhaps not surprisingly, that recovery after a major insult to the CNS is not necessarily a restoration of pre-injury properties, but a functional adaptation to a “new normal” [37].

### 2.2. Neurotrophins

Neurotrophins, such as brain-derived neurotrophic factor (BDNF), glial-cell-derived neurotrophic factor (GDNF), and Neurotrophins 3 and 4 (NT-3 and NT-4, respectively), are a class of growth factors that promote neurogenesis, neuroregulation, synaptic regeneration, neuroprotection, and neuronal survival [38,39,40]. BDNF and its high affinity receptor, TrkB, often dominate the spotlight, as countless studies have identified them as key players in many SCI sequelae, as well as in the pathways associated with exercise-based recovery (Figure 1B).

BDNF was shown to be regulated in a neural activity-dependent manner [41], and it has been known since the 1990s that physical activity in general, and the resultant neuronal activity, enhances the expression of BDNF in the CNS in animal models [42]. While some molecular markers of plasticity are modulated in a task-dependent manner [43], BDNF is increased whether the rehabilitation program incorporates conventional treadmill training [18,44,45,46], water treadmill training [47], passive bicycling [44], wheel running [19], or swimming [45], regardless of sex and severity of injury. In humans, the results have become equally convincing, with even short bouts of exercise increasing BDNF serum levels in non-injured individuals and individuals with SCI alike [48,49,50].

SCI induces an abrupt decrease in activity of neural networks, which leads to reduced BDNF in the lumbar spinal cord below the injury [19,23,45,51,52] (Table 1). By repetitively activating the relevant neural networks, exercise promotes BDNF mRNA and protein expression in muscles [53] and throughout the length of the spinal cord [18,19,23,44,45,47,51,52,54]. This increase in BDNF promotes plasticity, bolsters the effect of descending drive on motoneurons, and helps normalize motoneuronal properties [21,55,56]. Consequently, the exercise-associated increase in BDNF is accompanied by multiple forms of functional recovery, including improved locomotor performance and decreased spasticity and allodynia [23,44,46,56]. The variety of sensorimotor functional improvements associated with the activity-dependent increase in BDNF after SCI likely stems from the complex and widespread influence that BDNF has on numerous biological functions, including axon regeneration, synaptic plasticity, myelination, neuronal survival, and reduced inflammation (reviewed in [56,57]), and its complex interplay with different neurotransmitter cascades [58]. This versatility is perhaps due to the diverse molecular reach of BDNF’s high-affinity receptor, TrkB. Once BDNF binds to the TrkB receptor, three major intracellular signaling cascades ensue: the PI3-K/Akt pathway, the PLC/PKC pathway, and the Ras/ERK pathway, all of which modulate neuronal survival, growth, and plasticity to varying degrees with exercise following SCI. This can be inferred by BDNF-associated increases in synaptic markers [18,19], inhibitory neurotransmitters [23], and transcription factors [19,23,47] (Figure 1B).

The BDNF/TrkB pathway is known to increase both synthesis and phosphorylation of Synapsin I to trigger the MAPK signaling pathway and modulate neurotransmitter release in cortical neurons [59]. It is believed that the exercise-induced increase in BDNF may account for the observed increase in Synapsin I phosphorylation in SCI rats [19], as previously reported in the hippocampus [60]. While many experiments have highlighted a correlation between increases in BDNF and exercise-based recovery, a growing number of studies have confirmed its necessary and causal role in the recovery processes. When TrkB-IgG, a molecule that competes with endogenous TrkB and thus sequesters BDNF, is delivered to animals undergoing rehabilitation, the exercise-induced improvements in allodynia [46], locomotor impairment [61], and spasticity [62] are eliminated. The activity-dependent increase in BDNF/TrkB is also associated with an increase in synthesis of the transcription factor cyclic AMP response element binding protein (CREB) and p-CREB, its phosphorylated form, in the spinal cord caudal to the lesion [19,23]. Accordingly, blocking TrkB signaling after SCI significantly reduces CREB and p-CREB and prevents activity-dependent functional recovery [23,47]. Together, these studies unequivocally identify BDNF-TrkB as a crucial agent of exercise-mediated signaling.

In the hippocampus [63], prolonged exercise was found to induce an accumulation of the metabolite β-hydroxybutyrate (BHB) in the liver. BHB crosses the blood–brain barrier into hippocampal neurons, in which it inhibits the class I histone deacetylases HDAC2 and HDAC3, which act upon selective BDNF promoters. In doing so, BHB specifically promotes BDNF expression via Ca^2+^-dependent epigenetic modifications [64,65,66]. While it is plausible that a similar mechanism exists in the spinal cord, and a class I HDAC inhibitor was recently shown to induce modest locomotor improvements in SCI mice [67], a direct link between exercise-induced metabolites and BDNF has yet to be demonstrated in an SCI model.

While BDNF signaling responds to exercise most prominently, other neurotrophins, such as NT-3 [19,44,45,52,68], NT-4 [44,69], NGF, and GDNF [44,53], are also linked with exercise-induced recovery after SCI. However, these growth factors are less frequently studied following SCI, and their contribution to recovery remains incompletely understood. Nonetheless, there is overwhelming evidence suggesting that exercise increases the expression of various neurotrophins after SCI, and that it contributes to facilitate recovery. This is further supported by experiments in which exogenous neurotrophins are provided and contribute to functional recovery [57,70,71,72,73]. However, increasing neurotrophins, particularly BDNF, after SCI is not likely the perfect remedy for SCI patients. Exogenous delivery of BDNF can be accompanied by debilitating side effects, such as neuropathic pain and hyperreflexia [57,72,74,75,76]. While the intensity, duration, and type of exercise yields differential increases of BDNF in the spinal cord of animals with SCI [19,45,77,78] and in blood serum in humans [50], it appears that exercise does not produce BDNF in detrimental excess. This suggests that exercise has a homeostatic effect, and to date, exercise is considered the safest method of increasing neurotrophin expression.

### 2.3. Serotonin Receptors

Serotonin (5-HT) is a vital neurotransmitter in the mammalian spinal cord, playing an essential role in modulating sensorimotor function. In the intact spinal cord, serotoninergic innervation is almost entirely derived from the raphe nuclei in the brainstem, which is lost [79,80] or severely compromised following SCI [81]. The drastic and sudden decrease in serotonin availability below the lesion renders spinal networks unexcitable and unresponsive [82,83], which critically contributes to SCI-induced paralysis [80]. The importance of 5-HT to movement is exemplified by the restoration of neuronal excitability and associated locomotor behaviors observed when 5-HT or 5-HT receptor agonists are administered, both in the acute or chronic phase of injury (reviewed in [84]). However, while spinal 5-HT availability is undeniably decreased after SCI, the serotonergic system remains challenging to study compared to other systems, as it comprises intraspinal and supraspinal neurons, as well as numerous receptors, all of which are highly plastic.

The serotoninergic system exerts its function via an interaction between 5-HT and a number of 5-HT receptors expressed in the spinal cord. Following SCI, the loss of descending 5-HT supply causes 5-HT receptors to undergo varying degrees of plasticity, depending on the specific subtype. Here, we will mainly focus on the receptors that have been clearly demonstrated to undergo anatomical and/or functional changes following injury and impact motor output.

Considerable interest has been devoted to 5-HT_1A_, 5-HT_2A_, and 5-HT_2C_ in terms of their anatomical and functional changes, likely due to their direct involvement in regulating motoneuronal excitability and functional recovery after SCI [80,85]. In the chronic phase of SCI, a number of 5-HT receptors are upregulated in the spinal cord. Most notably 5-HT_1A_ [35,86], 5-HT_2A_ [35,87,88,89,90,91,92], and 5-HT_2C_ [79,81,93] (Table 1). This increase in receptor expression perhaps represents the development of a compensatory mechanism to counterbalance the lack of 5-HT. This is supported by the fact that (1) motoneurons become extremely sensitive even to small amounts of 5-HT [82]; (2) pharmacologically activating 5-HT receptors after SCI with quipazine (5-HT_2A/2C_R agonist) and 8-OH-DPAT (5-HT_1A/7_R agonist) can prevent the upregulation of 5-HT_1A/2A_ receptors below the lesion [35]; and (3) 5-HT_2C_R becomes constitutively active (active in the absence of 5-HT) after chronic SCI in rat motoneurons, which contributes to the recovery of locomotion [79,94]. Interestingly, while 5-_HT1A/2A/2C_R are increased after injury, exercise does not decrease their expression toward intact levels [35,87] to restore motor function as it does for inhibitory markers (see Section 2.4) and BDNF (Section 2.2). This is likely because 5-HT is not produced in the spinal cord. Instead, exercise further enhances the expression of 5-HT_2A_R (Figure 1B), a change that is specifically observed in extensor motoneurons [87].

These studies have led to the use of Buspirone, a 5-HT_1A/2/7_R agonist, in human subjects with SCI to enhance spinal cord excitability and promote locomotor recovery, either alone or in combination with activity-based interventions such as treadmill training [95,96,97] or other pharmacological agents [98]. Interestingly, the presence of sensory feedback from the limbs is critical to the upregulation of 5-HT_1A_R, as it does not occur in deafferented animals after SCI [86]. Since the presence of proprioceptive feedback is essential to motor recovery [99], this further supports a role for limb afferents to enhance 5-HT receptor expression in response to exercise. In addition, while exercise does not prevent 5-_HT1/2_R upregulation, combining passive bicycling with 5-HT agonists both prevented the upregulation and improved locomotor recovery [35]. This is in agreement with other studies demonstrating that although 5-HT agonists improve functional recovery, the approach is more successful when combined with exercise [36,100,101], suggesting a synergistic effect. However, the potential to translate this to the clinic remains unclear, as 5-HT agonists also trigger undesirable effects and contribute to psychiatric disorders [102] and pain [103].

A lot remains to be determined about the intricacies of how exercise affects the different facets of serotoninergic pathways after SCI, as the effect of exercise has not been investigated in regard to many 5-HT receptor subtypes. However, our current knowledge suggests that they have an important role in promoting activity-dependent neural plasticity.

### 2.4. Inhibitory Neurotransmitters

GABA and glycine are the chief inhibitory neurotransmitters in the central nervous system, making their synthesis and release important determinants of neuronal processing. Disruptions in GABAergic and glycinergic signaling have widespread functional effects, which range from schizophrenia to depression, epilepsy, and neuropathic pain [104,105]. After SCI, the loss of supraspinal descending input also results in alterations in inhibitory synaptic transmission. A number of factors contribute to this, including increases in (1) the size and density of presynaptic inhibitory inputs; (2) postsynaptic expression of glycinergic (GlyR) and GABA_A_ receptors (GABA_A_R); (3) expression of the anchoring protein gephyrin, a peptide closely associated with GlyR; and (4) the two GABA synthetizing enzymes, glutamic acid decarboxylase 65 and 67 (GAD_65_ and GAD_67_, respectively) in the spinal segments below the lesion [37,106,107,108,109].

Exercise has been shown to reverse/prevent the disruptions in GABAergic and glycinergic signaling in several pathologies, including epilepsy [110], neuropathic pain [111,112], anxiety [113], and also SCI. Specifically, step-training was shown to decrease GAD_67_, GlyR, and GABA_A_R expression in the lumbar spinal cord toward intact levels after injury [37,43,107] (Table 1). Interestingly, stand-training did not decrease the same markers of inhibition, hinting at the importance of providing appropriate proprioceptive feedback to restore spinal inhibition. While this reflects general changes in inhibitory signaling that arise after SCI, recent studies have suggested that plasticity in this pathway is more complex than initially thought. For example, a month following a contusive SCI, the expression of both GAD_65_ and GAD_67_ was reported to be decreased in the dorsal horn, rather than increased, with treadmill training reversing this effect, likely via BDNF-TrkB dependent pathways [23] (Figure 1B). Multiple factors may contribute to the discrepancies between studies, including the animal model, injury type/severity, and time after injury, but also the differences in where and how GAD_67/65_ was quantified. This is supported by a recent study in which GAD_65_ expression was increased in axon terminals on motoneuron somata, but decreased in presynaptic boutons contacting primary afferents [36,114]. By taking into account not only the overall expression of inhibitory markers, but also their density, size, and location, exercise was revealed to increase both the density and size of GAD_65_-positive presynaptic boutons (Figure 1A), suggesting an increase in modulation of afferent input, which perhaps contributes to the restoration of presynaptic inhibition [115]. In contrast, GAD_65_-positive axon terminals contacting motoneuron somata increased in number with exercise but shrank in size. Similarly, exercised animals displayed an increased number of glycinergic terminals contacting motoneuron somata, each of smaller size than those found in animals with SCI (Figure 1A). This suggests either sprouting of existing axon terminals, or newly formed connections from new/spared GABAergic and glycinergic interneurons. Interestingly, these exercise-induced changes were accompanied by significant recovery of locomotor ability, but differed substantially from the connections observed in intact animals. In fact, this was exemplified by the emergence of a new stepping pattern observed only in SCI-exercised rats [36]. This demonstrates that recovery does not necessarily involve a restoration of pre-injury properties, but a functional adaptation to new conditions [37].

While separate studies may have indicated opposite effects of injury on GAD_67/65_ expression, exercise was shown to counteract the changes, suggesting that exercise may have a homeostatic effect. This may occur via changes in BDNF-TrkB signaling, as BDNF modulates GAD_65_ and GAD_67_ expression following SCI [23] (Figure 1B). This is in agreement with the dual role of BDNF in preventing or promoting downstream pathways depending on the surrounding cellular environment [73,116] (further discussed in Section 2.5).

### 2.5. Chloride Homeostasis

The alterations in inhibitory synaptic transmission after SCI are not only due to a general change in markers of inhibition (Section 2.4), but also to changes in the cation chloride co-transporters KCC2 (and/or NKCC1) that regulate intracellular chloride levels ([Cl^−^]_i_) required for inhibition [36,51,73,87,117,118,119]. [Cl^−^]_i_ is largely governed by the cation chloride cotransporters KCC2 (chloride extruder) and NKCC1 (chloride intruder) [120]. Their relative expression determines the chloride reversal potential, and thus regulates the amplitude and polarity of GABAergic and glycinergic responses. After SCI, there is a sudden and severe reduction in excitability of neurons below the lesion, due in part to the lack of serotonergic neuromodulation that normally emanates from descending tracts. This causes the sub-lesional cord to recapitulate a state observed during early development: KCC2 is downregulated, Cl^−^ ions accumulate within the neurons, and GABA- and glycine-mediated responses are less hyperpolarizing, and can even be depolarizing [73,121] (Figure 1B). The importance of this shift is revealed by its involvement in the development of both spasticity and neuropathic pain after SCIs [73,122,123].

Exercise improves these symptoms in the clinic [124,125], and recent studies from animal models of SCI suggest that these improvements are associated with an increase in expression of KCC2 around motoneurons below the lesion [20,36,46,51,62,87]. Moreover, KCC2 was identified as both necessary and sufficient for these effects. Exercise-induced improvements in spasticity are mimicked by a single dose of a KCC2-enhancing compound in unexercised rats with SCI [126], and are eliminated when KCC2 activity is blocked during exercise [62]. This effect was found to rely on BDNF/TrkB signaling, as chelating BDNF with TrkB-IgG during exercise prevented both the upregulation of KCC2 and the exercise-dependent functional improvements [46,62]. This provides evidence that beyond modulating classical synaptic plasticity, the activity-dependent increase in BDNF is also a robust mediator of ionic plasticity.

Importantly, the regulation of KCC2 by BDNF changes following SCI, with BDNF promoting KCC2 expression after injury, rather than downregulating it [73,116]. Potential mechanisms driving this change remain to be fully explored, but a likely possibility is the contribution of PLCγ (Phospholipase C) and Shc [127,128,129]. BDNF has been shown to induce an increase in Shc and downregulate KCC2 when PLC-γ is present, but upregulate KCC2 when PLC-γ is absent [127,128]. Fittingly, expression of PLC-γ is reduced within 14 days following SCI [46] (Figure 1B). While exercise increases the expression of BDNF, it does not impact the expression of PLC-γ (Table 1), and therefore leads to KCC2 upregulation [46]. Interestingly, the increase in KCC2 with exercise is also paralleled by a significant preservation of spinal PNNs [20]. The proteoglycans found in PNNs can contribute to chloride homeostasis and the polarity of GABA signaling [130]. The simultaneous increase in KCC2 expression on motoneurons and increase in synthesis of PNN components during development [131,132] suggests a potential relationship between these markers that can be reversed by exercise. Together, this suggests that chloride homeostasis may play an important role in the homeostatic effects observed with exercise.

Additionally, 5-HT_2A_R have also been implicated in the regulation of KCC2 in the spinal cord [73]. After SCI, the pharmacological activation of 5-HT_2A_R, which hyperpolarizes the reversal potential of inhibitory postsynaptic potentials (IPSPs), increases cell membrane expression of KCC2 and restores endogenous inhibition [117]. While some studies have found exercise to increase the expression of the 5-HT_2A_R following SCI [35,87], whether this contributes to the upregulation of KCC2 in synergy with the BDNF pathway remains to be determined.

## 3. Rehabilitation Promotes Sprouting and Regeneration

Beyond synaptogenesis and the strengthening of existing pathways, anatomical plasticity in response to exercise after SCI also includes axonal growth, regeneration through the core of the lesion, sprouting of spared neural tissue, and the formation of novel relays. The remodeling of spinal cord connectivity can also be triggered by increased sprouting and plasticity of axonal connections.

### 3.1. Serotoninergic Fiber Sprouting

Injury-induced sprouting has been shown to be enhanced by exercise such as treadmill training or wheel running [17,27,133,134,135,136]. However, very little is known about the signaling pathways contributing to these changes, as they have mostly been identified in terms of which supraspinal pathway is involved. Due to its vital role in supraspinal modulation, serotonin is often a focus of these studies.

Beyond changes in the expression of spinal 5-HT receptors induced by exercise *(*Section 2.3*)*, supraspinal serotoninergic projections that are spared after an incomplete SCI can also undergo plastic changes. Serotoninergic projections originating from the raphe nuclei extend to motoneurons in the ventral horn of the spinal cord to regulate their activity. It has been long known that increasing serotonergic activity with exogenous serotonergic agonists facilitates stepping after SCI in animal models [137,138,139], and that the presence of 5-HT fibers and terminals in the spinal cord and the recovery of locomotor function after injury is strongly correlated [140,141,142,143]. Exercise was shown to increase (1) 5-HT fiber outgrowth, (2) the number of 5-HT fibers, and (3) the amount of presynaptic terminals around motoneurons in the ventral horn caudal to an incomplete injury (Figure 1A). In agreement with earlier studies, this was associated with functional recovery, including improvements in locomotion and reaching/grasping [22,133,136]. Although the causality of this relationship remains to be determined, this suggests that exercise can promote axonal sprouting to supplement the loss of 5-HT supply and improve function. Potential mechanisms for this likely involve exercise-induced increases in BDNF, as it is implicated in structural changes in the lumbar spinal cord following SCI, including increased sprouting of serotonergic axons [133].

### 3.2. Markers of Regeneration

Perhaps the most obvious approach to treat SCI is to reestablish communication between the separated segments of the CNS. Axonal regeneration was long considered the gold standard to cure SCI by achieving regrowth across the lesion site. However, several decades of research have proven it is not a trivial endeavor [144]. In that context, exercise is mostly used to guide regenerating axons towards creating functional circuits. This is a critical step, as without appropriate guidance, newly formed connections are likely to reach inappropriate targets and lead to maladaptive plasticity and aberrant function [145,146].

A very limited number of studies have focused on the effect of exercise alone on regeneration, perhaps because early studies suggested that exercise only modestly impacts axon regeneration across the lesion site and might not be sufficient to induce robust recovery [147,148,149]. However, any treatment aiming to be translatable to the clinic needs to include a rehabilitation program, as it is incorporated in the standard of care. In any case, there is now a consensus that an effective treatment for SCI will require the inclusion of activity-based therapy to promote functional recovery and effectively enhance neuroplasticity [15,147,150]. Most often, exercise is combined with pharmacological and/or surgical approaches to manipulate neuronal intrinsic growth programs that encourage axon regeneration. These include peripheral nerve grafts (PNG) [148,151], neural stem-cell grafts [149,152], and enzymes that degrade inhibitors of axon regeneration [27,153,154], all of which contribute to establishing a growth and plasticity permissive environment [148,149,150,151].

To date, there are no convincing data supporting a role for exercise in increasing the regenerative potential and improving function in SCI models of regeneration [155,156,157,158]. However, a number of anatomical changes were identified. For example, cycling exercise has been shown to increase propriospinal regeneration into a PNG and modulate the expression of genes associated with regeneration, including mRNA levels for GAP43 and β-actin, both involved in axon elongation, and neuritin, which promotes synapse formation and neurite outgrowth [148] (Figure 1C). Similarly, combining neural stem-cell grafts with treadmill training significantly improved regenerative capability after contusion injury in mice by increasing the number of Elavl^+^ cells, suggesting that exercise facilitated neuronal differentiation within the stem-cell graft [149]. The addition of treadmill training also increased the immunoreactivity of pGAP43 in the spinal cord caudal to the injury site, and was accompanied by a significant increase in other molecules that regulate synaptic plasticity, such as VGlut1, GAD_65_, and Synapsin I, as well as functional improvements in reflex inhibition. While this is encouraging, results still remain limited in terms of functional recovery, especially after chronic SCI [149,152].

### 3.3. Bypassing the Lesion

Long descending supraspinal tracts are particularly susceptible to damage by an SCI. As such, a substantial amount of supraspinal rewiring takes place at a distance from the lesion, and it is now well known that this is important for recovery [159,160,161,162]. There are several detour pathways that have been shown to relay information from supraspinal locomotor commands through the lesion site after incomplete SCI. Amongst these pathways, the contribution of exercise to the corticospino-propriospinal pathway [159] and the corticospino-reticulospinal pathway [163] are the best described.

In rats [159] and in humans [164], combining electrical stimulation of the spinal cord with locomotor training improves voluntary control of overground stepping even when stimulation is discontinued, suggesting that adding training to the neuromodulatory treatment is essential to shape the circuitry in a meaningful manner [159]. These new circuits arise when collaterals of transected corticospinal fibers migrate into the grey matter to contact neurons of either the long propriospinal tract [136,159] or the reticulospinal tract [163]. These subsequently bypass the lesion site to contact lumbar motoneurons, and form cortically controlled detour pathways [136,165]. Even without the addition of neuromodulatory treatments, rehabilitation provided via irregular running wheels was shown to increase formation of these circuits up to threefold [136] (Figure 1C). The rewiring persisted for many weeks and was accompanied by improvements in skilled motor tasks, which depend on corticospinal input. Interestingly, uninjured animals that underwent the same rehabilitative program showed no such changes in circuitry, suggesting that this effect of exercise on circuit plasticity is specific to damaged pathways [136].

## 4. Other Considerations

Beyond the damage incurred by the lesion itself, known as the primary mechanical injury, there are further mechanisms of damage that follow. Inflammation, neural and glial cell death, and maladaptive immune responses all contribute to what is known as the secondary injury, which leads to expansion of the initial damage to more distal segments of the cord [166]. Although most of these factors are studied in terms of their contribution to lesion size and neuropathic pain after SCI, which is not the focus of this review, they also have the potential to affect motor recovery. By destroying the blood–spinal-cord barrier, an SCI allows blood-borne neutrophils to flood into the lesion site. This triggers a near-immediate upsurge in inflammatory mediators, such as TNF-α and IL-1β, as well as activation of microglia [167,168] (Figure 1C).

Inflammation after SCI induces an abundant number of complications, including activation of multiple apoptotic signaling proteins and the subsequent death of neurons and oligodendrocytes (demyelination) [168]. Several studies have found that exercise reduces expression of inflammatory markers after SCI in animal models [169,170] and in humans [171], but how this occurs has yet to be clearly delineated. While there are numerous mechanisms likely at play, one possibility is via an increase in vascular endothelial growth factor (VEGF). VEGF promotes endothelial cell proliferation, vascularization, and angiogenesis, and is upregulated by exercise via lactate receptors expressed in the blood–brain barrier [172]. After SCI, the exercise-induced increase in VEGF is associated with enhanced expression of tight junctions and adherens junctions, which reduces blood–spinal-cord barrier permeability and improves locomotor recovery [173]. This effect was further shown to rely on BDNF/TrkB signaling [47], as well as inhibition of matrix metalloproteinases (MMPs) [173], which restructure extracellular matrices and damage the blood–spinal-cord barrier after SCI [174,175]. Interestingly, another study found that after SCI, proliferating endothelial cells located in the core of the lesion upregulate CD200, which hinders inflammation [176], while yet another study found that treadmill exercise upregulates both CD200 and its receptor after stroke in rats [177]. Thus, it is possible that the decrease in inflammatory signaling observed with exercise occurs in part by increasing the endothelial release of CD200 and increasing the expression of the CD200 receptor. However, this has yet to be investigated in a model of SCI.

The local responses of glial cells, astrocytes and oligodendrocytes in particular, are also a critical determinant of motor recovery, as they contribute to the non-permissible environment limiting axon regeneration [178]. Rehabilitation significantly decreases glial fibrillary acidic protein (GFAP)^+^ astrogliosis, i.e., reactive astrocytes and their deposition of extracellular matrix molecules, such as CSPGs, both in the epicenter and in the spinal cord caudal to the lesion [22,136] (Figure 1C). As astrogliosis inhibits axonal growth through the formation of the glial scar after SCI, this suggests that rehabilitation can modify the injury environment and improve functional recovery. Similarly, it was found that exercise reduces microglia activation in the spinal cord [179], though there remains substantial ambiguity surrounding microglia and their effect on motor recovery.

## 5. Potential for Translation and Limitations

Following SCI, exercise-based therapies are associated with countless forms of plasticity, ranging from circuit formation, prevention of apoptosis, axonal sprouting, changes in chloride homeostasis, and many other alterations that likely contribute to neural repair and functional recovery. While the mechanisms responsible for this activity-dependent plasticity remain to be completely described, there is substantial evidence implicating the upregulation of neurotrophins as a cardinal regulator of these processes.

As a result of its versatility and non-invasive nature, exercise is routinely used in the clinic as a strategy to improve motor recovery. While there is abundant evidence supporting improved functional recovery and quality of life with exercise and activity-based therapies after SCI in humans, the number of randomized controlled clinical trials remains limited, and shows considerable variability across studies and patients in response to therapy [180,181]. Amongst the critical limitations of clinical trials is the need to standardize therapy, while in the clinic, activity-based therapies need to be highly individualized, both in modality and intensity based on specific needs to optimize functional improvements. In addition, the many co-morbidities that accompany SCI can make exercise challenging or even impossible for some individuals, particularly early after injury. Thus, there remains a need for pharmacological therapies that can be combined with exercise or substituted for exercise when necessary. Caution is warranted, however, as pharmacological interventions produce systemic changes in signaling, unlike exercise, which, by inducing neural activity in relevant networks, alters molecular pathways in a more finely tuned, spatio-temporal manner.

Nonetheless, it is now widely accepted that to significantly regain motor function after an SCI, it will necessitate a combination of cellular and pharmacological treatments that address the variety of targets and processes affected by the SCI, and that these approaches are more robust when combined with activity-based therapies [15,182]. Optimizing these strategies will critically rely on a firm understanding of the molecular pathways involved in recovery, many of which were identified by investigating the effect of activity-based therapies in animal models of SCI. Finally, future therapeutic developments would benefit greatly from bench studies that consider the injury model and severity, the type of exercise provided, appropriate timing for initiation, as well as the spinal region of interest.

**Table 1:** Table summarizing key molecular changes in the spinal cord below the lesion after SCI and with exercise.

Section	Chronic SCI	References	Chronic SCI + Exercise	References
**2.1. Synaptic Plasticity**	↓ CREB	[19,23]	↑ CREB	[19,23,54]
	↓ Synapsin I	[19,54]	↑ Synapsin I	[19,22,54]
	↓ Synaptophysin	[18,19,183]	↑ Synaptophysin	[17,28]
	↓ PSD-95	[17]	↑ PSD-95	[17]
	↓ PNN	[20,27]	↑ PNN	[20,27]
**2.2. Neurotrophins**	↓ BDNF	[18,19,23,45,51,54]	↑ BDNF	[18,19,23,44,45,46,47,51,54]
**2.3. Serotonin Receptors**	↑ 5-HT_1A_	[35,86]	↑ 5-HT_1A_	[35]
	↑ 5-HT_2A_	[35,87,88,89,90,91,92]	↑ 5-HT_2A_	[35,87]
	↑ 5-HT_2C_	[79,81,93]	= 5-HT_2C_	[87]
**2.4. Markers of in inhibition**	↑↓ GlyR	↑ [37] = [36] ↓ [114]	↓↑ GlyR	↓ [37] ↑ [36]
	↑↓ GABA_A_R	↑↓ [107] ↓ [114]	↓↑ GABA_A_R	↑↓ [107]
	↑↓ GAD_67_	↑ [106] ↓ [23]	↓↑ GAD_67_	[25,45]
	↑↓ GAD_65_	= [36,106] ↓ [23]	↑↓ GAD_65_	↑ [23] = [43] ↑↓ [36]
**2.5. Chloride Homeostasis**	↓ KCC2	[20,36,51,62,73,87]	↑ KCC2	[20,36,46,51,62,87]
	↓ PLCγ	[46]	= PLCγ	[46]

Legend: ↑ increased, ↓ decreased, = no change, ↑↓ increased or decreased depending on location.

## Data Availability

Not applicable.
